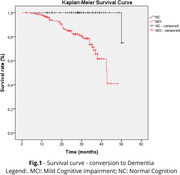# Conversion to dementia up to the fourth annual follow‐up assessment and from a Brazilian cohort of older adults

**DOI:** 10.1002/alz.093477

**Published:** 2025-01-09

**Authors:** Gabriela Tomé Oliveira Engelmann, Carolina Portugal Vieira, Ivonne Carolina Bolaños Burgos, Giovanna Correia Pereira Moro, João Marcos Silva Borges, Julia Cardoso Costa Cardoso Costa, Guilherme Fonseca Graciano, Aline Siqueira de Souza, Marcelle Ferreira Saldanha, Erika de Oliveira Hansen, Marco Aurélio Romano‐Silva, Debora Marques de Miranda, Luiz Armando Cunha de Marco, Rafaela Teixeira de Ávila, Bernardo de Mattos Viana, Maria Aparecida Camargos Bicalho

**Affiliations:** ^1^ Cog‐Aging Research Group, Universidade Federal de Minas Gerais (UFMG), Belo Horizonte, Minas Gerais Brazil; ^2^ Jenny de Andrade Faria Institute – Outpatient Reference Center for the Elderly, Universidade Federal de Minas Gerais (UFMG), Belo Horizonte, Minas Gerais Brazil; ^3^ Molecular Medicine Postgraduate Program, School of Medicine, Universidade Federal de Minas Gerais (UFMG), Belo Horizonte, Minas Gerais Brazil; ^4^ Molecular Medicine Program, Faculdade de Medicina, Belo Horizonte Brazil; ^5^ Undergraduate Medicine, Federal University of Minas Gerais, Belo Horizonte, Minas Gerais Brazil; ^6^ Sciences Applied to Adult Health Postgraduate Program, School of Medicine, Universidade Federal de Minas Gerais (UFMG), Belo Horizonte, Minas Gerais Brazil; ^7^ Neurotec R National Institute of Science and Technology (INCT‐Neurotec R), Faculty of Medicine, Universidade Federal de Minas Gerais (UFMG), Belo Horizonte, Minas Gerais Brazil; ^8^ Older Adult’s Psychiatry and Psychology Extension Program (PROEPSI), School of Medicine, Universidade Federal de Minas Gerais (UFMG), Belo Horizonte, Minas Gerais Brazil; ^9^ undergraduate Medicine, Federal University of Minas Gerais, Belo Horizonte, Minas Gerais Brazil; ^10^ Cog‐Aging Group Research, Brazil, Belo Horizonte, Minas Gerais Brazil; ^11^ Federal University of Minas Gerais, Belo Horizonte, Minas Gerais Brazil; ^12^ Cog‐Aging Research Group, Belo Horizonte, Minas Gerais Brazil; ^13^ Department of Psychiatry, School of Medicine, Federal University of Minas Gerais, Belo Horizonte, Minas Gerais Brazil; ^14^ Molecular Medicine Postgraduate Program, Faculty of Medicine, Universidade Federal de Minas Gerais (UFMG, Belo Horizonte, Minas Gerais Brazil; ^15^ Molecular Medicine Postgraduate Program, Faculty of Medicine, Universidade Federal de Minas Gerais (UFMG), Belo Horizonte, Minas Gerais Brazil; ^16^ Universidade Federal de Minas Gerais, Belo Horizonte Brazil; ^17^ FUMEC, Belo Horizonte Brazil; ^18^ Department of Psychiatry, School of Medicine, Universidade Federal de Minas Gerais (UFMG), Belo Horizonte, Minas Gerais Brazil; ^19^ Department of Clinical Medicine, Faculty of Medicine, Universidade Federal de Minas Gerais (UFMG), Belo Horizonte, Minas Gerais Brazil

## Abstract

**Background:**

Studies comprising cohorts of cognitively unimpaired older adults or with mild cognitive impairment (MCI) that assesses clinical and biological characteristics to conversion to dementia are scarce in Brazil.

**Method:**

To determine the median conversion time to dementia in a cohort of Brazilian older adults with low educational levels. In the cohort, participants undergo an annual comprehensive clinical and neuropsychological evaluation, complemented by neuroimaging and laboratory assessments to determine the diagnosis. Data from 225 older adults from the Cog‐Aging cohort was assessed and the first confirmed diagnosis was selected for this analysis: 67 Normal Cognition (NC) and 158 with MCI. Descriptive statistics were carried out according to normality tests. The time to conversion to dementia for participants with normal cognition (NC), Mild Cognitive Impairment (MCI) was assessed using the Kaplan‐Meier survival analysis. Only p<0.05 were considered. This study was approved by UFMG’s ethics committee.

**Result:**

The median age at the beginning of the study was 73 years (IQR = 9.19) for NC group, and 77 years for MCI (IQR = 10.58). The median years for education was 4 years for both the NC (IQR = 4.50) and MCI (IQR = 2.00) groups. At the fourth annual follow‐up, 66 patients with NC remained stable, while 21 with MCI remitted to NC. The cumulative proportion surviving of MCI patients was 68,7% (SE .054) at 36 months, with 31 conversions to dementia and 37 remaining cases. At 36 months, no NC had converted to dementia. This occurred at 50 months. The median survival time was 42.74 months (SE 2.92; Lower Bound 37.01; Upper 48.48). In other terms, this means that 50% of MCI conversions to dementia occurrs up to 3.56 years. These differences were statistically significant with Log Rank (Mantel‐cox), Breslow (Generalized Wilcoxon) and Tarone‐Ware p≤ .001.

**Conclusion:**

At the fourth annual follow‐up assessment, patients with NC tend to remain stable, while 50% of participants with MCI convert to dementia up to 3.56 years. MCI poses a greater risk for conversion to dementia in the first’s three years of follow‐up in this sample, however more studies are still needed to assess other confounding variables.